# Effects of different mechanisms on antimicrobial resistance in *Pseudomonas aeruginosa*: a strategic system for evaluating antibiotics against gram-negative bacteria

**DOI:** 10.1128/spectrum.02418-24

**Published:** 2025-03-05

**Authors:** Yu-Kuo Tsai, Jen-Chang Chang, Jia-Je Li, Esther Yip-Mei Liu, Chang-Phone Fung, Ching-Hsun Wang, Feng-Yee Chang, Jung-Chung Lin, L. Kristopher Siu

**Affiliations:** 1 KeMyth Biotech Company, Incubation Center, National Health Research Institutes, Miaoli, Taiwan; 2 National Institute of Infectious Diseases and Vaccinology, National Health Research Institutes, Miaoli, Taiwan; 3 Division of Infectious Diseases and Tropical Medicine, Department of Internal Medicine, Tri-Service General Hospital, National Defense Medical Center, Taipei, Taiwan; 4 Division of Infectious Diseases, Cathay General Hospital, New Taipei City, Taiwan; 5 Institute of Microbiology and Immunology, National Defense Medical Center, Taipei, Taiwan; 6 Graduate Institute of Basic Medical Science, China Medical University, Taichung, Taiwan; ICON plc, London, United Kingdom

**Keywords:** multidrug-resistant bacteria, antibiotic development, resistance mechanisms, *Pseudomonas aeruginosa*, gram-negative bacteria

## Abstract

Our previous studies constructed a strategic system for testing antibiotics against specific resistance mechanisms using *Klebsiella pneumoniae* and *Acinetobacter baumannii*. However, it lacked resistance mechanisms specifically expressed only in *Pseudomonas* species. In this study, we constructed this system using *Pseudomonas aeruginosa*. In-frame deletion, site-directed mutagenesis, and plasmid transformation were used to generate genetically engineered strains with various resistance mechanisms from two fully susceptible *P. aeruginosa* strains. Antimicrobial susceptibility testing was used to test the efficacy of antibiotics against these strains in vitro. A total of 31 engineered strains with various antimicrobial resistance mechanisms from *P. aeruginosa* KPA888 and ATCC 27853 were constructed, and the same antibiotic resistance mechanism showed a similar effect on the MICs of the two strains. Compared to the parental strains, the engineered strains lacking porin OprD or lacking the regulator genes of efflux pumps all showed a ≥4-fold increase on the MICs of some of the 19 antibiotics tested. Mechanisms due to GyrA/ParC mutations and β-lactamases also contributed to their corresponding resistance as previously published. The strains constructed in this study possess well-defined resistance mechanisms and can be used to screen and evaluate the effectiveness of antibiotics against specific resistance mechanisms in *P. aeruginosa*. Building upon our previous studies on *K. pneumoniae* and *A. baumannii*, this strategic system, including a *P. aeruginosa* panel, has been expanded to cover almost all the important antibiotic resistance mechanisms of gram-negative bacteria that are in urgent need of new antibiotics.

**IMPORTANCE:**

In this study, an antibiotic assessment system for *P. aeruginosa* was developed, and the system can be expanded to include other key pathogens and resistance mechanisms. This system offers several benefits: (i) compound design: aid in the development of compounds that can bypass or counteract resistance mechanisms, leading to more effective treatments against specific resistant strains; (ii) combination therapies: facilitate the exploration of combination therapies, where multiple antibiotics may work synergistically to overcome resistance and enhance treatment efficacy; and (iii) targeted treatments: enable healthcare providers to prescribe more targeted treatments, reducing unnecessary antibiotic use and helping to slow the spread of antibiotic resistance. In summary, this system could streamline the development process, reduce costs, increase the success rate of new antibiotics, and help prevent and control antimicrobial resistance.

## INTRODUCTION

Antibiotic resistance is becoming a chronic pandemic problem to bacterial infections worldwide. In 2016, the Drugs for Neglected Diseases *initiative* and the World Health Organization (WHO) have established the Global Antibiotic Research and Development Partnership to accelerate the development of new antibiotics to treat multidrug-resistant bacterial infections (1). In 2017 and 2024, the WHO published a list of bacteria currently posing the greatest threat, among which *Acinetobacter baumannii*, *Pseudomonas aeruginosa*, and Enterobacterales (especially *Klebsiella pneumoniae*, *Escherichia coli*, *Salmonella*, and *Shigella*) belong to both lists (2, 3). These bacteria are of particular concern because they can cause severe infections and are becoming increasingly difficult to treat with existing antibiotics.

Developing new antibiotics against gram-negative bacteria is a challenging task due to their complex and efficient resistance mechanisms. The traditional approach uses a large number of clinical isolates to test the effectiveness of new antibiotics. However, the resistance mechanisms of these isolates usually are not fully characterized, and their genetic backgrounds almost are different. Unknown factors may be involved in these resistant bacteria and make it difficult to verify which mechanism plays the major role in conferring drug resistance. To address this issue, our previous studies used fully susceptible clinical *K. pneumoniae* and *A. baumannii* isolates as parental strains to construct mutants with different resistance mechanisms (4, 5). These mutants have been used to test compounds targeting specific resistance mechanisms and facilitate antibiotic development, such as sulbactam–durlobactam (6).

*P. aeruginosa* is one of the most common pathogenic bacteria causing infections and an important cause of morbidity and mortality worldwide (7, 8). The emergence of multidrug-resistant strains of *P. aeruginosa* is increasing and difficult to treat (2, 9), and the slow pace of new antibiotic development exacerbates the challenge (8, 10). In the present work, the same various mutants with different resistance mechanisms were constructed from two fully susceptible *P. aeruginosa* strains, and the effects of each resistance mechanism on the two strains were compared. The chromosomally mediated resistance mechanisms that were constructed are permeability defects generated by deletion of the gene encoding the outer-membrane proteins OprD, OprO, OprP, OpdD, OpdF, OpdK, or OpdO (11, 12), efflux pump overexpression generated by deletion of the regulatory gene *mexR*, *nalC*, *nalD*, *nfxB*, *mexS*, or *mexZ* of the efflux pump MexAB-OprM, MexCD-OprJ, MexEF-OprN, or MexXY-OprM (or -OprA) (12–15), and modification of quinolone target sites via GyrA and/or ParC mutations (12, 13, 16). Most plasmid-mediated mechanisms are specific to one antibiotic group, and this study focused on the β-lactam antibiotics (17–22). This study aimed to construct a strategic system for screening and evaluating compounds against *Pseudomonas* species.

## RESULTS AND DISCUSSION

### Porin loss in antibiotic resistance

Porins can facilitate the passage of small hydrophilic antibiotics through the outer membrane of gram-negative bacteria (23). Previous studies have shown that the outer membrane permeability of *P. aeruginosa* is about 12–100-fold less than that of *E. coli* (24, 25), and the reasons include the lack of unspecific porins, which facilitate the diffusion of small hydrophilic molecules across the outer membrane (26). In this study, the deletion of OprD resulted in a ≥4-fold increase in the MICs of imipenem and meropenem, whereas the deletion of other outer membrane porins had minimal (<2-fold) effects on the MICs of all antibiotics tested (Table 1). This is consistent with the results reported for another clinical isolate of *P. aeruginosa* (27), whose antimicrobial susceptibility data showed that of the 40 identifiable porins in this isolate, only carbapenems could partially enter through OprD (27). However, when these porins are combined with other resistance mechanisms, their effects on the MICs may require further studies.

**TABLE 1:** MICs of antibiotics against chromosomally mediated resistance mechanisms, including loss of OprD, OprO, OprP, OpdD, OpdF, OpdK, or OpdO porins in *P. aeruginosa* KPA888 and ATCC 27853

Parental strain and mutation^*a*^	MIC (mg/L)^*b*^																		
	ATM^*c*^	PIP	CAZ	CZA	FEP	C/T	IMI	MRP	CIP	LEV	AK	CN	TOB	TE	TGC	CS	FOS	C	SXT
KPA888																			
None	1	1.5	0.75	0.75	1	0.38	1	0.25	0.064	0.25	3	1.5	1	12	8	3	4	32	1
∆*oprD*	1	1.5	0.75	0.5	1	0.25	**6**	**2**	0.064	0.25	3	1.5	1	12	8	4	4	32	1.5
∆*oprO*	1.5	2	1	0.75	1	0.38	1	0.19	0.064	0.25	3	1.5	1.5	12	8	4	4	32	1.5
∆*oprP*	1	1.5	0.75	0.5	0.75	0.38	0.75	0.19	0.094	0.38	3	1.5	0.75	12	8	3	3	32	1
∆*opdD*	1	1.5	0.75	0.5	1	0.38	0.75	0.25	0.094	0.38	3	1.5	1	12	8	4	4	32	1.5
∆*opdF*	1	2	0.75	0.75	1	0.5	1	0.25	0.094	0.25	3	1.5	1	12	8	3	4	32	1.5
∆*opdK*	1	2	0.75	0.75	1	0.38	1	0.25	0.094	0.38	3	1.5	1.5	12	8	4	3	32	1.5
∆*opdO*	1.5	1.5	0.75	0.75	1	0.38	0.75	0.25	0.094	0.25	3	1.5	1	12	8	3	4	32	1.5
ATCC 27853																			
None	3	2	1	1	2	0.75	2	0.38	0.19	0.75	4	1.5	0.75	24	12	2	6	>256	>32
∆*oprD*	4	3	1	1	3	0.75	**16**	**4**	0.19	1	6	1.5	0.75	32	12	3	8	>256	>32
∆*oprO*	3	3	1.5	1	3	0.75	2	0.5	0.19	1	4	1.5	0.75	24	12	3	8	>256	>32
∆*oprP*	4	2	1.5	1	2	0.75	2	0.5	0.19	1	4	1.5	0.75	32	12	2	6	>256	>32
∆*opdD*	3	3	1	1	3	0.75	2	0.5	0.19	0.75	6	1.5	0.75	24	12	2	8	>256	>32
∆*opdF*	3	3	1.5	1	2	0.75	3	0.5	0.19	0.75	4	1.5	0.75	24	12	2	8	>256	>32
∆*opdK*	3	3	1.5	1	2	0.75	2	0.5	0.19	1	4	1.5	0.75	32	12	2	6	>256	>32
∆*opdO*	3	3	1.5	1	3	0.75	2	0.38	0.19	1	4	1.5	0.75	24	12	2	6	>256	>32

^*a*^ The mutation constructed in the genes in *P. aeruginosa* KPA888 or ATCC 27853 is shown.

^*b*^ The boldface numbers indicate a ≥4-fold shift in the MICs of the parental strain (*P. aeruginosa* KPA888 or ATCC 27853) and its derived mutants. The MIC data represent results from at least two independent experiments, both of which showed identical or similar results (<2-fold difference).

^*c*^ ATM, aztreonam; PIP, piperacillin; CAZ, ceftazidime; CZA, ceftazidime/avibactam with a fixed concentration of 4 mg/L; FEP, cefepime; C/T, ceftolozane/tazobactam with a fixed concentration of 4 mg/L; IMI, imipenem; MRP, meropenem; CIP, ciprofloxacin; LEV, levofloxacin; AK, amikacin; CN, gentamicin; TOB, tobramycin; TE, tetracycline; TGC, tigecycline; CS, colistin; FOS, fosfomycin; C, chloramphenicol; SXT, trimethoprim/sulfamethoxazole (only the trimethoprim portion of the 1/19 drug ratio is displayed).

### Efflux pumps in antibiotic resistance

In *P. aeruginosa*, overexpression of four main sets of efflux pumps plays a crucial role in drug resistance, namely, MexAB-OprM, MexCD-OprJ, MexEF-OprN, and MexXY-OprM (or -OprA) (12, 15), and the mutation of their regulatory genes can lead to overexpression (13, 14). In this study, overexpression of the four efflux pumps was achieved by deleting their regulatory genes. Different resistance profiles were found (Table 2), and all of the four efflux pumps can result in a ≥4-fold increase in the MICs of quinolones and chloramphenicol, whereas all showed minimal (<2-fold) effects on the MICs of colistin and fosfomycin as previously described (13, 15). Regarding the MexAB-OprM efflux pump, ≥4-fold increases in the MICs were found for β-lactams, quinolones, tetracyclines, chloramphenicol, and/or trimethoprim/sulfamethoxazole when comparing the MICs of the Δ*mexR*, Δ*nalC*, and Δ*nalD* mutants to the parental strain (Table 2). In the MexCD-OprJ efflux pump, when comparing the MICs of the ∆*nfxB* mutants to the parental strain, ≥4-fold increases in the MICs were found for cefepime, quinolones, and/or chloramphenicol, while 2- to 3-fold shifts were found for the MICs of tetracyclines (Table 2). Regarding the MexEF-OprN efflux pump, both Δ*mexS* mutants had higher MICs for imipenem and meropenem than the parental strain (Table 2), which may be due to the deletion of *mexS* reducing the transcript level of the *oprD* gene (28), a specific porin, which can facilitate the uptake of carbapenems (27). In the MexXY-OprM (or -OprA) efflux pump, the ∆*mexZ* mutants were more resistant than the parental strain to cefepime, quinolones, aminoglycosides, and/or chloramphenicol, and the MICs of these antibiotics shifted by at least 2-fold (Table 2).

**TABLE 2:** MICs of antibiotics against chromosomally mediated resistance mechanisms, including loss of the regulatory genes of the efflux pumps MexAB-OprM, MexCD-OprJ, MexEF-OprN, or MexXY-OprM (or -OprA) in *P. aeruginosa* KPA888 and ATCC 27853

Parental strain and mutation^*a*^	MIC (mg/L)^*b*^																		
	ATM^*c*^	PIP	CAZ	CZA	FEP	C/T	IMI	MRP	CIP	LEV	AK	CN	TOB	TE	TGC	CS	FOS	C	SXT
KPA888																			
None	1	1.5	0.75	0.75	1	0.38	0.75	0.25	0.064	0.25	3	1.5	1	12	8	3	4	32	1
∆*mexR*	**8**	**8**	1.5	1.5	2	0.5	0.75	0.5	**0.25**	**1.5**	2	1.5	1	32	24	2	3	**>256**	**6**
∆*nalC*	**6**	**6**	1.5	1.5	3	0.5	1	0.5	**0.25**	**1**	3	1.5	0.75	32	24	2	4	**>256**	**6**
∆*nalD*	**6**	**8**	1.5	1.5	2	0.5	1	0.5	**0.25**	**1**	3	1.5	0.75	24	16	3	4	**>256**	**6**
∆*nfxB*	0.5	2	0.5	0.5	2	0.25	0.38	0.19	**0.5**	**2**	**0.75**	**0.38**	0.5	32	16	2	3	**>256**	1
∆*mexS*	0.38	0.5	0.5	0.5	0.75	0.38	2	0.5	0.19	0.75	1	0.5	0.5	16	16	2	3	**>256**	**>32**
∆*mexZ*	0.75	1	0.75	0.5	2	0.38	0.75	0.19	**0.25**	**1**	4	1.5	1	16	6	3	4	**>256**	1
ATCC 27853																			
None	3	2	1	1	2	0.75	2	0.38	0.19	0.75	4	1.5	0.75	24	12	2	6	>256	>32
∆*mexR*	**24**	**12**	3	3	**12**	1	2	**2**	0.5	**4**	4	1.5	0.75	**96**	**64**	2	8	>256	>32
∆*nalC*	**16**	**12**	2	2	**8**	1	2	**2**	0.5	**3**	3	1.5	0.75	**96**	**48**	2	6	>256	>32
∆*nalD*	**16**	**8**	3	2	6	0.75	2	**2**	0.5	**4**	3	1	0.75	48	32	2	8	>256	>32
∆*nfxB*	**0.75**	4	0.75	0.75	**8**	0.5	0.75	0.25	**0.75**	**24**	1.5	0.5	0.38	64	24	2	8	>256	>32
∆*mexS*	4	3	1.5	1	3	1	6	**2**	**0.75**	**8**	3	1.5	0.75	32	32	2	8	>256	>32
∆*mexZ*	3	3	1.5	1	**8**	1	2	0.38	0.38	**3**	8	3	2	32	16	2	8	>256	>32

^*a*^ The mutation constructed in the efflux pump genes or their regulatory genes in *P. aeruginosa* KPA888 or ATCC 27853 is shown.

^*b*^ The underlined numbers indicate a 2- to 3-fold shift in the MICs of the parental strain (*P. aeruginosa* KPA888 or ATCC 27853) and its derived mutants, while the boldface numbers indicate a ≥4-fold shift in the MICs. The MIC data represent results from at least two independent experiments, both of which showed identical or similar results (<2-fold difference).

^*c*^ cATM, aztreonam; PIP, piperacillin; CAZ, ceftazidime; CZA, ceftazidime/avibactam with a fixed concentration of 4 mg/L; FEP, cefepime; C/T, ceftolozane/tazobactam with a fixed concentration of 4 mg/L; IMI, imipenem; MRP, meropenem; CIP, ciprofloxacin; LEV, levofloxacin; AK, amikacin; CN, gentamicin; TOB, tobramycin; TE, tetracycline; TGC, tigecycline; CS, colistin; FOS, fosfomycin; C, chloramphenicol; SXT, trimethoprim/sulfamethoxazole (only the trimethoprim portion of the 1/19 drug ratio is displayed).

Collateral sensitivity refers to a phenomenon where the acquisition of resistance to one antibiotic results in increased sensitivity to other antibiotics (29). In this study, the ∆*nfxB* mutants showed an inherited collateral sensitivity to aztreonam, imipenem, and aminoglycosides tested (Table 2) as previously described (30). In addition, Hernando-Amado et al. have observed that in some clinical isolates of *P. aeruginosa* with different genomic backgrounds, the presence of ciprofloxacin can increase their resistance to ciprofloxacin while simultaneously increasing their susceptibility to aztreonam and tobramycin (31). By analyzing the entire genome sequences of these strains, it was found that mutations in *mexS* can cause this phenomenon because some strains only acquired mutations in this gene (31). In this study, similar results can also be found in the ∆*mexS* mutant of the KPA888 strain (Table 2). Research into the mechanisms of collateral sensitivity is ongoing, and a deeper understanding could lead to a more effective use of existing antibiotics and the development of new therapeutic strategies that exploit collateral sensitivity to manage antibiotic resistance (29, 32).

### Quinolone target site mutations in quinolone resistance

In the GyrA T83I mutant, the further ParC mutation (S87L or S87W) resulted in a ≥4-fold increase in the MICs of all the quinolones tested, whereas the further GyrA mutation (D87N) showed minimal (<2-fold) effects on these MICs (Table 3). In the T83I/S87L or T83I/S87W mutants, the further GyrA mutation (D87N) could increase these MICs to high-level resistance (Table 3). In all the quinolones tested, the single-mutation T83I or D87N in GyrA resulted in a ≥4-fold increase in MICs, whereas the single-mutation S87L or S87W in ParC showed minimal (<2-fold) effects on these MICs (Table 3). Interestingly, similar results were found in our previous studies constructing mutants with GyrA and/or ParC mutations in *K. pneumoniae* and *A. baumannii* (4, 5). This phenomenon may explain why GyrA mutations can be found in almost all clinical isolates of *P. aeruginosa* that are resistant to ciprofloxacin (MIC ≥ 2 mg/L), whereas no single parC mutation has been observed in these strains (16, 33, 34). On the contrary, Nouri et al. examined mutations in *gyrA* and *parC* genes in 64 *P*. *aeruginosa* clinical isolates with ciprofloxacin MICs ≥ 2 mg/L, where the T83I mutation in GyrA was found in all of these isolates (33). The average MIC of ciprofloxacin of these strains was T83I/D87N/S87L mutants > T83I/S87L mutants > T83I mutants (33), and our results are consistent with this finding.

**TABLE 3:** MICs of quinolones against chromosomally mediated resistance mechanisms, including GyrA and/or ParC mutations in *P. aeruginosa* KPA888 and ATCC 27853

Mutation^*a*^	MIC (mg/L)^*b*^											
	KPA888						ATCC 27853					
	NA^*c*^	CIP	NOR	OFX	LEV	GAT	NA	CIP	NOR	OFX	LEV	GAT
None	48	0.064	0.5	0.5	0.38	0.19	>256	0.19	1	1.5	0.75	0.5
GyrA T83I	**>256**	**0.75**	**3**	**4**	**4**	**1**	>256	**2**	**8**	**16**	**8**	**3**
GyrA D87N	**>256**	**0.5**	**3**	**3**	**1.5**	**0.75**	>256	**1**	**6**	**12**	**4**	**2**
ParC S87L	64	0.094	0.38	0.75	0.38	0.25	>256	0.19	1	1.5	1	0.5
ParC S87W	48	0.064	0.5	0.5	0.38	0.19	>256	0.19	1	1.5	0.75	0.5
GyrA T83I; ParC S87L	**>256**	**12**	**64**	**>32**	**16**	**8**	>256	**>32**	**128**	**>32**	**>32**	**12**
GyrA T83I; ParC S87W	**>256**	**12**	**64**	**>32**	**16**	**6**	>256	**>32**	**128**	**>32**	**>32**	**16**
GyrA D87N; ParC S87L	**>256**	**0.75**	**16**	**4**	**4**	**1.5**	>256	**3**	**32**	**16**	**8**	**4**
GyrA D87N; ParC S87W	**>256**	**0.75**	**12**	**4**	**4**	**1.5**	>256	**3**	**24**	**16**	**8**	**4**
GyrA T83I/D87N	**>256**	**0.75**	**3**	**4**	**3**	**1**	>256	**2**	**8**	**16**	**8**	**3**
GyrA T83I/D87N; ParC S87L	**>256**	**>32**	**>256**	**>32**	**>32**	**>32**	>256	**>32**	**>256**	**>32**	**>32**	**>32**
GyrA T83I/D87N; ParC S87W	**>256**	**>32**	**>256**	**>32**	**>32**	**>32**	>256	**>32**	**>256**	**>32**	**>32**	**>32**

^*a*^ The mutation constructed on the GyrA and/or ParC in *P. aeruginosa* KPA888 or ATCC 27853 is shown

^*b*^ The boldface numbers indicate a ≥4-fold shift in the MICs of the parental strain (*P. aeruginosa* KPA888 or ATCC 27853) and its derived mutants. The MIC data represent results from at least two independent experiments, both of which showed identical or similar results (<2-fold difference).

^*c*^ cNA, nalidixic acid; CIP, ciprofloxacin; NOR, norfloxacin; OFX, ofloxacin; LEV, levofloxacin; GAT, gatifloxacin.

### β-Lactams against the plasmid-mediated resistance mechanism

Comparing the MICs to the parental strain, all of the extended-spectrum β-lactamase (ESBL)-producing strains, including CTX-M-14, CTX-M-15, and VEB-3, exhibited ≥4-fold higher MICs on aztreonam and cefepime, and both of the AmpC β-lactamase-producing strains, including CMY-2, showed ≥4-fold higher MICs on piperacillin and ceftazidime (Table 4). All of the ESBL- or AmpC β-lactamases tested showed a <4-fold effect on the MICs of ceftazidime/avibactam, ertapenem, imipenem, meropenem, and doripenem (Table 4). Each carbapenemase tested, including KPC-2, NDM-1, and VIM-1, resulted in a ≥4-fold increase in the MICs of nearly all the antibiotics tested (Table 4). Minimal (<2-fold) effects on the MICs of ceftazidime/avibactam were observed for the KPC-2 strains (Table 4), as avibactam can restore the activity of ceftazidime by inhibiting the KPC family (35). Aztreonam is an effective antibiotic against NDM-1 and VIM-1 strains (Table 4) as previously described (36). However, since metallo-β-lactamase-producing strains often coproduce other β-lactamases that can hydrolyz aztreonam, combination therapy with aztreonam is considered a potential treatment for these infections (37, 38).

**TABLE 4:** MICs of β-lactams against plasmid-mediated resistance mechanisms, including extended-spectrum β-lactamase, AmpC β-lactamase, or carbapenemase in *P. aeruginosa* KPA888 and ATCC 27853

Parental strain and supplemental plasmid^*a*^	MIC (mg/L)^*b*^										
	ATM^*c*^	PIP	TZP	CAZ	CZA	FEP	C/T	ETP	IMI	MRP	DOR
KPA888											
None	1	1.5	2	0.75	0.75	1	0.38	2	0.75	0.25	0.19
pUCP30M	1.5	1.5	2	0.75	0.75	1	0.5	2	0.75	0.19	0.19
CTX-M-14	**6**	**48**	**12**	2	0.75	**48**	0.5	6	0.75	0.25	0.38
CTX-M-15	**32**	**96**	3	**8**	0.5	**96**	0.38	3	0.5	0.19	0.19
VEB-3	**192**	**8**	6	**>256**	1.5	**64**	**4**	4	0.75	0.25	0.19
CMY-2	3	**6**	4	**8**	0.75	2	0.5	4	1	0.25	0.38
KPC-2	**>1024**	**>256**	**>256**	**48**	1	**>256**	**16**	**>32**	**16**	**>32**	**>32**
NDM-1	1.5	**>256**	**>256**	**>256**	**>256**	**>256**	**>256**	**>32**	**32**	**>32**	**>32**
VIM-1	1	**>256**	**>256**	**>256**	**>256**	**>256**	**>256**	**>32**	**24**	**>32**	**>32**
ATCC 27853											
None	3	2	3	1	1	2	0.75	8	2	0.38	0.38
pUCP30M	3	3	3	1	1	1.5	0.5	8	2	0.38	0.25
CTX-M-14	**64**	**>256**	**32**	**8**	1	**>256**	0.75	24	2	0.75	0.75
CTX-M-15	**96**	**>256**	6	**24**	1	**>256**	0.75	8	1.5	0.5	0.5
VEB-3	**48**	6	2	**128**	0.75	**16**	2	8	2	0.38	0.38
CMY-2	8	**16**	**12**	**32**	1	4	0.75	16	2	0.38	0.25
KPC-2	**>1024**	**>256**	**>256**	**>256**	1	**>256**	**8**	**>32**	**>32**	**>32**	**>32**
NDM-1	3	**>256**	**>256**	**>256**	**>256**	**>256**	**>256**	**>32**	**>32**	**>32**	**>32**
VIM-1	3	**>256**	**>256**	**>256**	**>256**	**>256**	**>256**	**>32**	**>32**	**>32**	**>32**

^*a*^ The extended-spectrum β-lactamase, AmpC β-lactamase, or carbapenemase on the plasmid pUCP30M is shown, and the plasmid was transferred into *P. aeruginosa* KPA888 and ATCC 27853.

^*b*^ The boldface numbers indicate a ≥4-fold shift in the MICs of the parental strain (*P. aeruginosa* KPA888 or ATCC 27853) and its derived mutants, while the underlined numbers indicate a 2- to 3-fold shift in the MICs. The MIC data represent results from at least two independent experiments, both of which showed identical or similar results (<2-fold difference).

^*c*^ ATM, aztreonam; PIP, piperacillin; TZP, piperacillin/tazobactam with a fixed concentration of 4 mg/L; CAZ, ceftazidime; CZA, ceftazidime/avibactam with a fixed concentration of 4 mg/L; FEP, cefepime; C/T, ceftolozane/tazobactam with a fixed concentration of 4 mg/L; ETP, ertapenem; IMI, imipenem; MRP, meropenem; DOR, doripenem.

### Conclusion

The ESKAPE pathogens, including *Enterococcus faecium*, *Staphylococcus aureus*, *K. pneumoniae*, *A. baumannii*, *P. aeruginosa*, and *Enterobacter* species, are notorious for their antibiotic resistance (39, 40). Among them, *K. pneumoniae*, *A. baumannii*, and *P. aeruginosa* have also been recognized by the WHO as gram-negative bacteria urgently requiring new antibiotics (2, 3). This study constructed 31 genetically engineered strains with different antibiotic resistance mechanisms from fully susceptible clinical isolates *P. aeruginosa* KPA888 and ATCC 27853 , with similar effects on MICs observed for the same resistance mechanisms (Table 5). These strains have well-defined resistance mechanisms while remaining sensitive to certain antibiotics. Furthermore, non-conjugative plasmids without transposons were used to introduce plasmid-mediated resistance, reducing the risk of horizontal gene transfer and random integration into the host genome. These features make these strains safer than resistant clinical isolates for assessing the antibacterial potency of compounds. Our previously constructed antibiotic-resistant strains of *K. pneumoniae* and *A. baumannii* also share these characteristics (4, 5). Together, these panels provide a robust system to support the development of new antibiotics targeting gram-negative bacteria.

**TABLE 5:** Antibiotic resistance mechanisms in fully susceptible *P. aeruginosa* KPA888 and ATCC 27853

Strain or plasmid	Genotype or description^*a*^	Major substrate^*b*^
Strain with chromosomally mediated resistance mechanism		
∆*oprD* mutant	∆*oprD*	IMI, MRP
Δ*oprO* mutant	Δ*oprO*	None
∆*oprP* mutant	∆*oprP*	None
∆*opdD* mutant	∆*opdD*	None
∆*opdF* mutant	∆*opdF*	None
∆*opdK* mutant	∆*opdK*	None
∆*opdO* mutant	∆*opdO*	none
Δ*mexR* mutant	Δ*mexR*	ATM, PIP, FEP, MRP, CIP, LEV, TE, TGC, C, SXT
Δ*nalC* mutant	Δ*nalC*	ATM, PIP, FEP, MRP, CIP, LEV, TE, TGC, C, SXT
Δ*nalD* mutant	Δ*nalD*	ATM, PIP, MRP, CIP, LEV, C, SXT
Δ*nfxB* mutant	Δ*nfxB*	ATM, FEP, CIP, LEV, AK, CN, C
Δ*mexS* mutant	Δ*mexS*	MRP, CIP, LEV, C, SXT
Δ*mexZ* mutant	Δ*mexZ*	FEP, CIP, LEV, C
T83I mutant	GyrA T83I	NA, CIP, NOR, OFX, LEV, GAT
D87N mutant	GyrA D87N	NA, CIP, NOR, OFX, LEV, GAT
S87L mutant	ParC S87L	None
S87W mutant	ParC S87W	None
T83I/S87L mutant	GyrA T83I; ParC S87L	NA, CIP, NOR, OFX, LEV, GAT
T83I/S87W mutant	GyrA T83I; ParC S87W	NA, CIP, NOR, OFX, LEV, GAT
D87N/S87L mutant	GyrA D87N; ParC S87L	NA, CIP, NOR, OFX, LEV, GAT
D87N/S87W mutant	GyrA D87N; ParC S87W	NA, CIP, NOR, OFX, LEV, GAT
T83I/D87N mutant	GyrA T83I/D87N	NA, CIP, NOR, OFX, LEV, GAT
T83I/D87N/S87L mutant	GyrA T83I/D87N; ParC S87L	NA, CIP, NOR, OFX, LEV, GAT
T83I/D87N/S87W mutant	GyrA T83I/D87N; ParC S87W	NA, CIP, NOR, OFX, LEV, GAT
Plasmid with plasmid-mediated resistance mechanism		
p30M/CTX-M-14	*bla*_CTX-M-14_ cloned into pUCP30M	ATM, PIP, TZP, CAZ, FEP
p30M/CTX-M-15	*bla*_CTX-M-15_ cloned into pUCP30M	ATM, PIP, CAZ, FEP
p30M/VEB-3	*bla*_VEB-3_ cloned into pUCP30M	ATM, PIP, CAZ, FEP, C/T
p30M/CMY-2	*bla*_CMY-2_ cloned into pUCP30M	PIP, TZP, CAZ
p30M/KPC-2	*bla*_KPC-2_ cloned into pUCP30M	ATM, PIP, TZP, CAZ, FEP, C/T, ETP, IMI, MRP, DOR
p30M/NDM-1	*bla*_NDM-1_ cloned into pUCP30M	PIP, TZP, CAZ, CZA, FEP, C/T, ETP, IMI, MRP, DOR
p30M/VIM-1	*bla*_VIM-1_ cloned into pUCP30M	PIP, TZP, CAZ, CZA, FEP, C/T, ETP, IMI, MRP, DOR

^*a*^ Amino acid replacements of GyrA and/or ParC are listed; resistance gene of the plasmid-mediated mechanism was cloned into the shuttle vector pUCP30M, which carries a gentamicin resistance determinant.

^*b*^ Listed antibiotics indicate a ≥4-fold shift in the MICs of the parental strain (*P. aeruginosa* KPA888 or ATCC 27853) and its derived mutants. ATM, aztreonam; PIP, piperacillin; TZP, piperacillin/tazobactam with a fixed concentration of 4 mg/L; CAZ, ceftazidime; CZA, ceftazidime/avibactam with a fixed concentration of 4 mg/L; FEP, cefepime; C/T, ceftolozane/tazobactam with a fixed concentration of 4 mg/L; ETP, ertapenem; IMI, imipenem; MRP, meropenem; DOR, doripenem; NA, nalidixic acid; CIP, ciprofloxacin; NOR, norfloxacin; OFX, ofloxacin; LEV, levofloxacin; GAT, gatifloxacin; AK, amikacin; CN, gentamicin; TOB, tobramycin; TE, tetracycline; TGC, tigecycline; CS, colistin; FOS, fosfomycin; C, chloramphenicol; SXT, trimethoprim/sulfamethoxazole (only the trimethoprim portion of the 1/19 drug ratio is displayed).

## MATERIALS AND METHODS

### Bacterial strains, plasmids, and growth conditions

Two fully susceptible *P. aeruginosa* strains were used to construct mutants with antibiotic resistance mechanisms (Table 5), including the clinical isolate KPA888 from Taiwan and the reference strain ATCC 27853. The plasmid pUCP30T-enhanced cyan fluorescent protein (eCFP) obtained from Addgene (Cambridge, MA, USA) originally contained the gene encoding eCFP. This gene was excised, and a multiple cloning site was introduced in its place, resulting in the construction of plasmid pUCP30M. Plasmid pUCP30M is a shuttle vector capable of replicating in *P. aeruginosa* and *E. coli* and carries a gentamicin resistance determinant. This shuttle vector was used to clone the resistance genes of the plasmid-mediated resistance mechanisms in this study (Table 5). Unless otherwise noted, *P. aeruginosa* and *E. coli* strains were cultured at 37°C in Mueller–Hinton medium with appropriate antibiotics.

### In-frame deletion mutagenesis

The plasmid pUT-KB (4), which consists of an R6K origin of replication, a mobRP4 origin of transfer, a *sacB* gene, and a kanamycin-resistant determinant, was ligated with a gentamicin resistant determinant to generate plasmid pUT-GKB for constructing mutants. Plasmid pUT-GKB is a suicide vector containing a counterselection marker, *sacB*, which originates from *Bacillus subtilis* (41). When this gene is expressed on the integrated pUT-GKB, it confers a sucrose-sensitivity phenotype, which enables positive selection with sucrose to detect the loss of the vector.

Gene deletions in the *P. aeruginosa* KPA888 and ATCC 27853 strains were constructed via in-frame deletion mutagenesis (42). Briefly, two DNA fragments (approximately 1 kb in size) that flanked the regions to be deleted were amplified by PCR using specific primer pairs. The two gel-purified PCR products containing complementary ends were then mixed and amplified via overlap PCR (43, 44). The resulting PCR fragment (approximately 2 kb in size) was digested with restriction enzymes, and then cloned into pUT-GKB, which had been similarly digested. For homologous recombination, the gene-deletion constructs in pUT-GKB were transformed into *E. coli* S17-1 λ*pir* (45) via electroporation and mobilized into *P. aeruginosa* KPA888 and ATCC 27853 via conjugation. Single-crossover strains were selected from *Pseudomonas* isolation agar (Sigma-Aldrich) plates supplemented with gentamicin (15 mg/L), and the insertion of plasmids was verified via PCR. After incubation in 20 mL Luria–Bertani (LB) broth for 6 h in the absence of gentamicin at 30°C, the fully grown cultures were spread onto LB agar plates supplemented with 10% sucrose. After the double crossover occurred, the sucrose-resistant and gentamicin-susceptible colonies were selected, and the gene deletions were confirmed via PCR.

### Site-directed mutagenesis

DNA fragments of the *gyrA* and *parC* genes along with their flanking regions were amplified from *P. aeruginosa* KPA888 and ATCC 27853 using PCR with specific primer pairs, and the PCR products were cloned into plasmid pUT-GKB. The QuikChange Lightning Site-directed Mutagenesis Kit (Agilent Technologies, USA) was used to generate mutations in the *gyrA* and *parC* genes in the plasmids using the methods described by the manufacturer. For homologous recombination, plasmids containing mutations in the *gyrA* or *parC* gene were transformed into *E. coli* S17-1 λ*pir* (45) via electroporation and mobilized into *P. aeruginosa* KPA888 and ATCC 27853 via conjugation. Single-crossover strains were selected from *Pseudomonas* isolation agar (Sigma-Aldrich) plates supplemented with gentamicin (15 mg/L), and the insertion of plasmids was verified via PCR. After incubation in 20 mL LB broth for 6 h in the absence of gentamicin at 30°C, the fully grown cultures were spread onto LB agar plates supplemented with 10% sucrose. After the double crossover occurred, the sucrose-resistant and gentamicin-susceptible colonies were selected, and the gene mutations were confirmed via DNA sequencing.

### Plasmid construction and transformation

DNA fragments of the resistance genes along with their flanking regions were amplified from clinical plasmids via PCR with specific primer pairs. The resulting PCR fragments were digested, and then cloned into the plasmid pUCP30M. The resulting plasmids were then transformed into *P. aeruginosa* KPA888 and ATCC 27853 via electroporation (46). The recombinant bacteria were plated onto Mueller–Hinton agar plates containing gentamicin (15 mg/L), and the presence of the cloned gene was confirmed via PCR and DNA sequencing.

### Antimicrobial susceptibility testing

The MICs of antibiotics were determined using the MIC test strip (Liofilchem, Italy) according to the manufacturer’s instructions. *P. aeruginosa* ATCC 27853 and *E. coli* ATCC 25922 were used as the quality control (QC) strains, and data were included only if the QC results fell within the acceptable range specified by the manufacturer’s instructions (Liofilchem, Italy). The MIC data represent results from at least two independent experiments, both of which showed identical or similar results (<2-fold difference).
